# Heterotetramerization of Plant PIP1 and PIP2 Aquaporins Is an Evolutionary Ancient Feature to Guide PIP1 Plasma Membrane Localization and Function

**DOI:** 10.3389/fpls.2018.00382

**Published:** 2018-03-26

**Authors:** Manuela D. Bienert, Till A. Diehn, Nicolas Richet, François Chaumont, Gerd P. Bienert

**Affiliations:** ^1^Metalloid Transport Group, Department of Physiology and Cell Biology, Leibniz Institute of Plant Genetics and Crop Plant Research, Gatersleben, Germany; ^2^Institut des Sciences de la Vie, Université catholique de Louvain, Louvain-la-Neuve, Belgium

**Keywords:** plasma membrane intrinsic protein, aquaporin, water transport, heterotetramer, oligomerization, trafficking, hydrogen peroxide

## Abstract

Aquaporins (AQPs) are tetrameric channel proteins regulating the transmembrane flux of small uncharged solutes and in particular water in living organisms. In plants, members of the plasma membrane intrinsic protein (PIP) AQP subfamily are important for the maintenance of the plant water status through the control of cell and tissue hydraulics. The PIP subfamily is subdivided into two groups: PIP1 and PIP2 that exhibit different water-channel activities when expressed in *Xenopus* oocytes or yeast cells. Most PIP1 and PIP2 isoforms physically interact and assemble in heterotetramers to modulate their subcellular localization and channel activity when they are co-expressed in oocytes, yeasts, and plants. Whether the interaction between different PIPs is stochastic or controlled by cell regulatory processes is still unknown. Here, we analyzed the water transport activity and the subcellular localization behavior of the complete PIP subfamily (SmPIP1;1, SmPIP2;1, and SmPIP2;2) of the lycophyte *Selaginella moellendorffii* upon (co-)expression in yeast and *Xenopus* oocytes. As observed for most of the PIP1 and PIP2 isoforms in other species, SmPIP1;1 was retained in the ER while SmPIP2;1 was found in the plasma membrane but, upon co-expression, both isoforms were found in the plasma membrane, leading to a synergistic effect on the water membrane permeability. SmPIP2;2 behaves as a PIP1, being retained in the endoplasmic reticulum when expressed alone in oocytes or in yeasts. Interestingly, in contrast to the oocyte system, in yeasts no synergistic effect on the membrane permeability was observed upon SmPIP1;1/SmPIP2;1 co-expression. We also demonstrated that SmPIP2;1 is permeable to water and the signaling molecule hydrogen peroxide. Moreover, growth- and complementation assays in the yeast system showed that heteromerization in all possible SmPIP combinations did not modify the substrate specificity of the channels. These results suggest that the characteristics known for angiosperm PIP1 and PIP2 isoforms in terms of their water transport activity, trafficking, and interaction emerged already as early as in non-seed vascular plants. The existence and conservation of these characteristics may argue for the fact that PIP2s are indeed involved in the delivery of PIP1s to the plasma membrane and that the formation of functional heterotetramers is of biological relevance.

## Introduction

Aquaporins (AQPs) are transmembrane channel proteins that control the facilitated diffusion of water and other uncharged solutes such as glycerol, hydrogen peroxide, ammonia, small organic acids, urea, and metalloids (Chaumont and Tyerman, 2017). AQPs are essential for the maintenance of the water status, osmoregulation, signal transduction, detoxification processes, and the acquisition and translocation of nutrients in various organisms (Chaumont and Tyerman, 2017). Genomes of bacteria and mammals comprise in most cases 2–13 *AQP* genes. In plant genomes, at least 19 (in *Selaginella moellendorffii*) (Anderberg et al., 2012) and up to 140 (*Brassica napus*) (Kayum et al., 2017) *AQP* genes have been identified. The large number of plant isoforms makes them an interesting and challenging study target, regularly revealing new functions, properties, and regulations.

Based on their sequence evolution, AQPs are divided into two major clades, which also echo their contrasting substrate spectra: the orthodox AQPs act as channels for water and small solutes such as hydrogen peroxide or ammonia, while the aquaglyceroporins (GLPs) are responsible for the transport of more bulky solutes, such as glycerol or metalloids. In plants, five phylogenetically distinct AQP subfamilies, namely the nodulin26-like intrinsic proteins (NIPs), the plasma membrane intrinsic proteins (PIPs), the small basic intrinsic proteins (SIPs), the tonoplast intrinsic proteins (TIPs), and the as yet poorly characterized X intrinsic proteins (XIPs) (Chaumont et al., 2001; Johanson et al., 2001; Danielson and Johanson, 2008) cluster with the AQP clade. One plant AQP subfamily, namely the GlpF-like intrinsic proteins (GIPs), clusters with the GLP clade (Gustavsson et al., 2005). All AQPs consist of six transmembrane-spanning helices linked by loops of varying lengths and termini facing the cytosol. Various crystal structures as well as biochemical and imaging studies showed that AQPs assemble in cell membranes in tetramers of a highly conserved structure (Tani and Fujiyoshi, 2014; Kirscht et al., 2016). Functional units of plant AQPs of various subfamilies are in most cases homotetramers consisting of four identical monomers. TIPs and PIPs constitute, by now, exceptions inhering the ability to form functional heterotetramers (Harvengt et al., 2000; Murozuka et al., 2013). Understanding PIP hetero-oligomerization is of tremendous importance and is addressed by many studies.

As identified by sequence comparisons in and between plant species, PIPs constitute the most homogeneous and member-richest AQP subfamily (Anderberg et al., 2012). Interestingly, despite the large number of PIP isoforms per genome, there is a persistence of a high sequence identity across species, probably due to a high selective pressure. After their identification and the demonstration of their water transport activity, a combination of physiological and molecular genetic evidence led to the conclusion that PIPs play key roles in the regulation of water fluxes through plant tissues and therefore in plant water homeostasis (Shinozaki and Yamaguchi-Shinozaki, 1992; Kammerloher et al., 1994; Chaumont and Tyerman, 2014; Maurel et al., 2015). In addition, more recently, several PIP isoforms have been shown to facilitate the membrane diffusion of other small molecules such as glycerol (Biela et al., 1999), urea (Gaspar et al., 2003), boric acid (Dordas et al., 2000; Dordas and Brown, 2001; Fitzpatrick and Reid, 2009; Mosa et al., 2016), arsenous acid (Mosa et al., 2012), hydrogen peroxide (reviewed in Bienert and Chaumont, 2014; Bienert and Bienert, 2017; Rodrigues et al., 2017), carbon dioxide (CO_2_) (Uehlein et al., 2003; reviewed in Uehlein et al., 2017), and even cations (Byrt et al., 2017).

Plasma membrane intrinsic proteins from vascular and non-vascular plants are subdivided into two major groups namely PIP1s and PIP2s. While PIPs from algae form one group and cannot be associated with neither the PIP1 nor PIP2 group of land plant PIPs, the presence of two conserved intron positions supports a common origin and function of all PIP isoforms (Anderberg et al., 2011). PIP1 and PIP2 isoforms share about 80% amino acid identity. The main differences between both groups are found in the length and amino acid composition of the N- and C-termini and the loop A (Chaumont et al., 2001). PIP1s and PIP2s have different properties when being heterologously expressed in the *Xenopus laevis* oocytes or the yeast *Saccharomyces cerevisiae* in terms of water transport activity and subcellular localization (Yaneff et al., 2015; reviewed in Jozefkowicz et al., 2017). In general, only PIP2s are able to mediate significant transmembrane water fluxes while most PIP1 isoforms do not (Fetter et al., 2004; Sakurai et al., 2005; Bellati et al., 2010; reviewed in Yaneff et al., 2015), except of a very limited number of PIP1 isoforms (Kammerloher et al., 1994; Tournaire-Roux et al., 2003; Suga and Maeshima, 2004; Zhang et al., 2007). Most PIP1 channels fail to be targeted to the plasma membrane and reside in intracellular membranes, typically the endoplasmic reticulum (ER). The intracellular retention of PIP1s is either due to missing plasma membrane trafficking signals or to existing ER retention motifs (Chevalier and Chaumont, 2015). For instance, when heterologously expressed singly in either plant, oocyte, or yeast cells, maize ZmPIP1;2 is retained in intracellular membranes, while ZmPIP2;5 is targeted to the plasma membrane. Interestingly, when co-expressed, both proteins are found in the plasma membrane, due to a physical interaction within a heterotetramer (Fetter et al., 2004; Zelazny et al., 2007; Bienert et al., 2014; Berny et al., 2016). This regulation mechanism is common to PIP1/PIP2 pairs derived from a range of plant species such as tobacco, grapevine, strawberry, durum wheat, *Beta vulgaris*, and *Mimosa pudica* (Temmei et al., 2005; Mahdieh et al., 2008; Vandeleur et al., 2009; Alleva et al., 2010; Bellati et al., 2010; Otto et al., 2010; Ayadi et al., 2011). Moreover, it was demonstrated that a 3:1, 1:3, and 2:2 stoichiometry of PIP1 versus PIP2 monomers coexists in maize and beet PIP heterotetramers (Berny et al., 2016; Jozefkowicz et al., 2016). In comparison to a PIP1 or a PIP2 homotetramer, PIP1/PIP2 heterotetramerization in different proportions affects not only the membrane water permeability, but also the sensitivity of the PIPs to protons or mercury and the solute selectivity (Bellati et al., 2010; Otto et al., 2010; Bienert et al., 2012). Although the interaction of most studied PIP1/PIP2 pairs results in an increase in the water permeability in oocytes, this is not always the case. For example, co-expression of ZmPIP1;1 with ZmPIP2;5 does not result in a synergistic effect on the membrane permeability compared to the expression of ZmPIP2;5 alone (Fetter et al., 2004). Similar results were obtained for BvPIP2;1/BvPIP1;1, OsPIP2;3/OsPIP1;3, and PvPIP2;3/PvPIP1;1 (Zhou et al., 2007; Matsumoto et al., 2009; Jozefkowicz et al., 2013). Moreover, hetero-oligomerization of two isoforms belonging to the same group such as ZmPIP2;6/ZmPIP2;1 and ZmPIP1;1/ZmPIP1;2 is also observed (Fetter et al., 2004; Cavez et al., 2009). The large number of PIP isoforms and thereof the resulting large combinatory possibilities of PIP interactions constitute a challenge to understand the structural features and the regulatory mechanisms of the oligomerization process and, more importantly, its biological relevance. Using mutational, biochemical, and imaging approaches, single amino acid residues in loop A and transmembrane domains of PIPs have been identified to impact heterotetramerization and therefore the trafficking and/or functionality (Bienert et al., 2012; Jozefkowicz et al., 2013; Berny et al., 2016). Focusing on PIP1/PIP2 pairs, which are expressed in the same tissues, contributes to validate the physiological relevance of this hetero-oligomerization mechanism. An alternative way is to investigate plant species with a very limited amount of PIP genes.

Here, we investigate PIPs from *S. moellendorffii* to understand the functional evolution of plant PIPs and the physical interaction between PIP1 and PIP2 isoforms. The spike moss *S. moellendorffii* belongs to the lycophytes representing one of the oldest living lineages having evolved a vascular tissue with only a central vein in no true leaves (Banks, 2009). Lycophytes had their zenith in the flora on earth during the carboniferous period (Banks, 2009). Compared to other sequenced plant species *S. moellendorffii* has the advantage to encode only three PIP isoforms which are clearly assigned to the PIP1 (SmPIP1;1) and PIP2 (SmPIP2;1 and SmPIP2;2) groups occurring also in higher plants. The small number of isoforms allows to experimentally analyze all possible PIP interactions occurring in this plant species. Furthermore, the sequences of SmPIPs differ partly in amino acid residues, which have been identified to play a role in the physical interaction of PIP proteins. We addressed the question whether the PIP hetero- versus homotetramerization process and its impact on the subcellular trafficking and transport function is an ancient feature present in non-seed plants or whether heterotetramerization has evolved in higher, evolutionary more recent, plant species to face physiological demands. To this aim, we characterized the three SmPIP isoforms in different expression systems and showed that the ability of PIP1;1 and PIP2;1 to physically interact and modify channel trafficking and/or water transport activity is intrinsic to these SmPIP isoforms. This suggests that heterotetramerization of PIP1 and PIP2 isoforms already occurs in early vascular plants. We hypothesize that the preservation of diverse PIP1 and PIP2 isoforms throughout plant evolution is advantageous. Moreover, we show that SmPIP heterotetramerization does not modify the solute selectivity of these channels.

## Materials and Methods

### Cloning and Vector Construction

*SmPIP1;1, SmPIP2;1*, and *SmPIP2;2* cDNAs (Anderberg et al., 2012) were optimized for expression in yeast by adapting their codon usage (performed by GenScript, New York, NY, United States). Primers (Supplementary File 1) matching the *SmPIP* sequences were used to PCR amplify the *SmPIP* cDNA sequences and generate the respective *SmPIP* constructs (Supplementary File 1). PCR products were cloned in the USER-yeast expression vector pYeDP60u (Hamann and Møller, 2007) with either a uracil or leucine auxotrophic selection marker (pYeDP60u-ura and pYeDP60u-leu) using a uracil excision-based improved high-throughput USER cloning technique (Nour-Eldin et al., 2006). For localization analyses in yeast, N-terminal GFP-tagging of *SmPIP1;1, SmPIP2;1*, and *SmPIP2;2* was achieved by a USER cloning procedure into the yeast expression vector pRS426-pTPI-N-ter-GFPu. For the localization study *in planta SmPIP1;1, SmPIP2;1*, and *SmPIP2;2* cDNAs were cloned into the plant expression vectors pCAMBIA2300 35S N-ter mYFPu and pCAMBIA2300 35S N-ter mCFPu (Bienert et al., 2011). *SmPIP1;1, SmPIP2;1*, and *SmPIP2;2* cDNAs were cloned into the USER-compatible *X. laevis* expression vectors pNB1u and pNB1-YFPu containing the YFP gene (Nour-Eldin et al., 2006). All sequences were verified by sequencing. N-terminal “X”FP fusions were chosen, as this has been shown to neither affect PIP activity nor the ability to interact in *Xenopus* oocytes or plant cells (Fetter et al., 2004; Zelazny et al., 2007; Bienert et al., 2012, 2014).

### Phylogenetic Analysis

ClustalW (integrated in GENEIOUS PRO v6.1) was used to build multiple sequence alignments for PIP protein sequences. The Bayesian phylogenetic analyses were done by using MrBayes version 3.2. To align the amino acids, the best-fit model Cprev of amino acid substitution was selected by MrBayes. Two parallel Metropolis-coupled Monte-Carlo Markov chain analyses with four chains for 2 million generations were conducted within MrBayes. Every 500 generation trees were sampled and the convergence of the runs was assessed using the standard deviation of split frequencies set <0.01. The protein sequences used for the generation of the phylogenetic tree can be found in GenBank or Phytozome v12.1 data libraries under the following accession numbers: AtPIP1;1: CAB71073, AtPIP1;2: AAC28529, AtPIP1;3: AAF81320, AtPIP1;4: AAF02782, AtPIP1;5: CAA20461, ZmPIP1;1: X82633, ZmPIP1;2: AF131201, ZmPIP1;3: AF326487, ZmPIP1;4: AF326488, ZmPIP1;5: AF326489, ZmPIP1;6: AF326490, SmPIP1;1: XP_002980090, SmPIP2;1: XP_002994427, SmPIP2;2: XP_002971714.1, AtPIP2;1: CAB67649, AtPIP2;2: AAD18142, AtPIP2;3: AAD18141, AtPIP2;4: BAB09839, AtPIP2;5: CAB41102, AtPIP2;6: AAC79629, AtPIP2;7: CAA17774, AtPIP2;8: AAC64216, PpPIP1;1: Pp1s1_535V6.4, PpPIP1;2: Pp1s102_107V6.1, PpPIP1;3: Pp1s305_12V6.1, PpPIP2;1: Pp1s8_151V6.1, PpPIP2;2: Pp1s55_301V6.2, PpPIP2;3: Pp1s267_61V6.1, PpPIP2;4: Pp1s118_199V6.1, PpPIP3;1: Pp1s17_281V6.1, ZmPIP2;1: AF326491, ZmPIP2;2: AF326492, ZmPIP2;3: AF326493, ZmPIP2;4: AF326494, ZmPIP2;5: AF130975, ZmPIP2;6: AF326495, and ZmPIP2;7: AF326496. Sequences of CcPIP4;1 and CcPIP4;2 were extracted from Anderberg et al. (2011) (Electronic Supplementary Material: Additional File 2) and CcPIP4;2 was used to conduct an outgroup rooting of the phylogenetic tree.

### Yeast Strains

For the growth and transport assays, different *S. cerevisiae* deletion mutant strains were co-transformed with either two empty vectors pYeDP60u-ura and pYeDP60u-leu (negative control), the empty vector pYeDP60u-leu and a respective positive control gene in pYeDP60u-ura, or combinations of two vectors pYeDP60u-ura and pYeDP60u-leu, containing the respective *SmPIP* cDNAs. For the ammonia and hydrogen peroxide transport assay, a triple mep yeast strain [Δ*mep1-3* (-ura/-leu) (Anna Maria Marini’s lab collection)] and a human *AQP8* as a positive control were used. For urea and boric acid transport assay, the yeast strains *Δdur3*/YHL016c/Y00947 (EUROSCARF) and *N. tabacum XIP1;1α* were used. For water transport assays and the subcellular localization analyses, the wild-type *S. cerevisiae* BY4741/Y00000 (EUROSCARF) yeast was used.

### Growth Assays

Transformed yeast was selected on synthetic medium containing 2% glucose, 50 mM succinic acid/Tris base, pH 6.5, 0.17% yeast nitrogen base (YNB) without amino acids and ammonium (Difco), 2% proline, and 2% agar [for Δ*mep1-3* (-ura/-leu)] or 2% glucose, 50 mM succinic acid/Tris base, pH 5.5, 0.7% YNB without amino acids (Difco), and 2% agar (for Δ*dur3* and wild-type BY4741). For the ammonium and urea complementation growth assays, yeast cells were spotted on synthetic medium containing 2% galactose, 50 mM succinic acid/Tris base, pH 6.5, 0.17% YNB without amino acids and ammonium (Difco), 2% agar, and different concentrations of ammonium or urea, or 2% proline or 1 mM arginine as positive control, respectively. For the hydrogen peroxide and boric acid toxicity growth assays, 2% galactose, 50 mM succinic acid/Tris base, pH 5.5, 0.7% YNB without amino acids (Difco), and different concentrations of hydrogen peroxide or boric acid were used. All media were supplemented according to the auxotrophic requirements with histidine, leucine, and methionine. Yeast cells were diluted to different OD_600_ values (1, 0.01, and 0.0001). Growth was documented after 7–10 days at 30°C.

### Water Transport Assay

*Saccharomyces cerevisiae* wild-type strain BY4741/Y00000 (EUROSCARF) was transformed with either two empty vectors pYeDP60u-ura and pYeDP60u-leu, the empty vector pYeDP60u-leu and hAQP8 in pYeDP60u-ura, or combinations of two vectors containing *SmPIP* cDNAs (one in pYeDP60u-ura and the other in pYeDP60u-leu or one in pRS426-pTPI-N-ter-GFPu and the other in pYeDP60u-leu). pRS426-pTPI-N-ter-GFPu is a yeast expression vector driving the expression of GFP fused to the N-terminus of indicated SmPIPs. Water transport assays were in principal performed as described in Medraño-Fernandez et al. (2016) and with the following modifications. Transformants were selected on synthetic medium (2% agar, 2% glucose, 50 mM succinic acid/Tris base, pH 5.5, and 0.7% YNB without amino acids supplemented according to the auxotrophic requirements with histidine and methionine). Transformants were grown in 4 ml synthetic medium for 12 h at 30°C and then transferred to 25 ml of synthetic medium (2% glucose replaced by 2% galactose) for 36 h at 30°C. After centrifugation, cells were resuspended in 3 ml of 50 mM KH_2_PO_4_ (pH 7.2) plus 6 μl of 2-mercaptoethanol, and incubated for 15 min at 30°C. Six milliliters of spheroplasting buffer [2.4 M sorbitol, 50 mM KH_2_PO_4_ (pH 7.2)] was supplied with 200 mg bovine serum albumin (Sigma) and 10 mg of Zymolyase 20T (Amsbio). The cells were incubated for 60 min at 30°C and 100 rpm. Following centrifugation, spheroplasts were washed once and finally resuspended in 10 mM Tris/MES, pH 8.0, 5 mM CaCl_2_, 50 mM NaCl, and 1.8 M sorbitol at an OD_600_ of 1.5. Kinetics of spheroplast swelling was measured as follows. Cell osmotic water permeability was measured after exposing spheroplasts to hypoosmotic conditions (transfer from 1.8 to 1.2 M sorbitol buffer). Volume changes were recorded via light scattering at an angle of 90° and 450 nm using a fast kinetics instrument (SFM-3000; BioLogic) at 25°C with a dead time of 1.5 ms. The time course of swelling was measured for 8 s at the acquisition rate of one measurement/0.0005 s. All data presented are averages of 22–28 trace recordings. Rate constants were calculated by fitting the traces using non-linear regression using GraphPadPrism6.0, as described previously (Liu et al., 2006). One-phase decay functions or two-phase decay functions were used to obtain the best fit of the swelling curves. Rate constants were calculated based on the best fit (Supplementary File 3). Three independent experiments were performed, all with consisting results.

### Subcellular Localization and Confocal Microscopic Imaging

Oocytes expressing YFP-tagged SmPIP constructs were prepared for microscopy as described by Sayers et al. (1997). Confocal images of oocyte cells were acquired 3 days after injection using a Zeiss 710 confocal microscope (Carl Zeiss, Jena, Germany). The YFP was excited with the 514 nm line of an argon multilaser and the emitted YFP fluorescence was detected between 530 and 570 nm. A Plan-Neofluar 10×/0.30 objective was used. Localization analysis has been performed twice with independent oocyte batches injected with independently generated cRNAs. Three to seven oocytes per injection event have been studied. Subcellular localization of YFP-tagged SmPIPs was identical in both experiments. Representative images are shown.

Transient expression of N-terminal mYFP- or mCFP-tagged SmPIP proteins in *Nicotiana benthamiana* abaxial epidermis cells of 3–5-week-old plants was done by syringe infiltration of transformed *Agrobacterium tumefaciens* strains (GV3101; co-infiltrated with a strain transformed with the P19 silencing repressor gene). For plasma membrane localization, the *Agrobacterium* cultures were washed with infiltration buffer (10 mM MES pH 5.6, 10 mM MgCl_2_). Thereafter, the cells were suspended in infiltration buffer, left on ice for 1 h, and then supplemented with 100 μM acetosyringone and 0.02% Tween 20, as Tween 20 was shown to enhance transformation efficiency (Zhao et al., 2017). Confocal images of transfected cells were acquired 2–4 days after transfection. Tobacco leaves were syringe-infiltrated with the plasma membrane marker FM4-64 (Molecular Probes – Life Technologies) at a concentration of 16 μM 10 min prior to imaging. FM4-64 was excited at 514 nm and detected from 640 to 760 nm. The YFP was excited with the 514 nm line of an argon multilaser and the emitted YFP fluorescence was detected between 517 and 552 nm. The CFP was excited with the 458 nm line of an argon multilaser and the emitted CFP fluorescence was detected between 450 and 514 nm. The localization was detected using a Zeiss LSM 780 confocal laser scanning microscope (Carl Zeiss, Jena, Germany). Three to four independent leave transformation events have been analyzed for each construct or combination of constructs. Transformation rates and efficiencies vary between experiments and infiltration events. Cells which co-express two SmPIPs are not observed at high frequency. At least 20 cells per transformation event have been analyzed. Representative images of subcellular expression patterns are shown.

BY4741 yeast cells expressing the GFP:PIP fusion protein alone or together with another non-tagged SmPIP were grown on SG medium without uracil but supplemented with histidine, leucine, and methionine according to the auxotrophic requirements (single expression) or without uracil and leucine but supplemented with histidine and methionine according to the auxotrophic requirements (co-expression). During the exponential growth phase (OD_600_ = 1–1.3), the localization was detected with a Zeiss LSM 780 confocal laser scanning microscope (Carl Zeiss, Jena, Germany) using a 488 nm laser line for excitation. GFP signals were detected at 491–530 nm. Two independent yeast transformation events per construct or combination of constructs have been performed to analyze the subcellular localization of SmPIPs. Subcellular localization pattern did not differ between the experiments. Representative images of subcellular SmPIP expression patterns are shown.

### *In Vitro* RNA Synthesis and Oocyte Transport Assays

Ready-to-use capped complementary RNAs encoding N-terminal YFP-tagged or non-tagged SmPIPs were synthesized *in vitro* as described previously (Fetter et al., 2004). *X. laevis* oocytes were isolated, defolliculated, and injected, and the osmotic water permeability coefficient (P_f_) determined as described previously (Fetter et al., 2004).

## Results

### *S. moellendorffii* PIPs Phylogenetically Cluster With PIP1s and PIP2s

**FIGURE 1:**
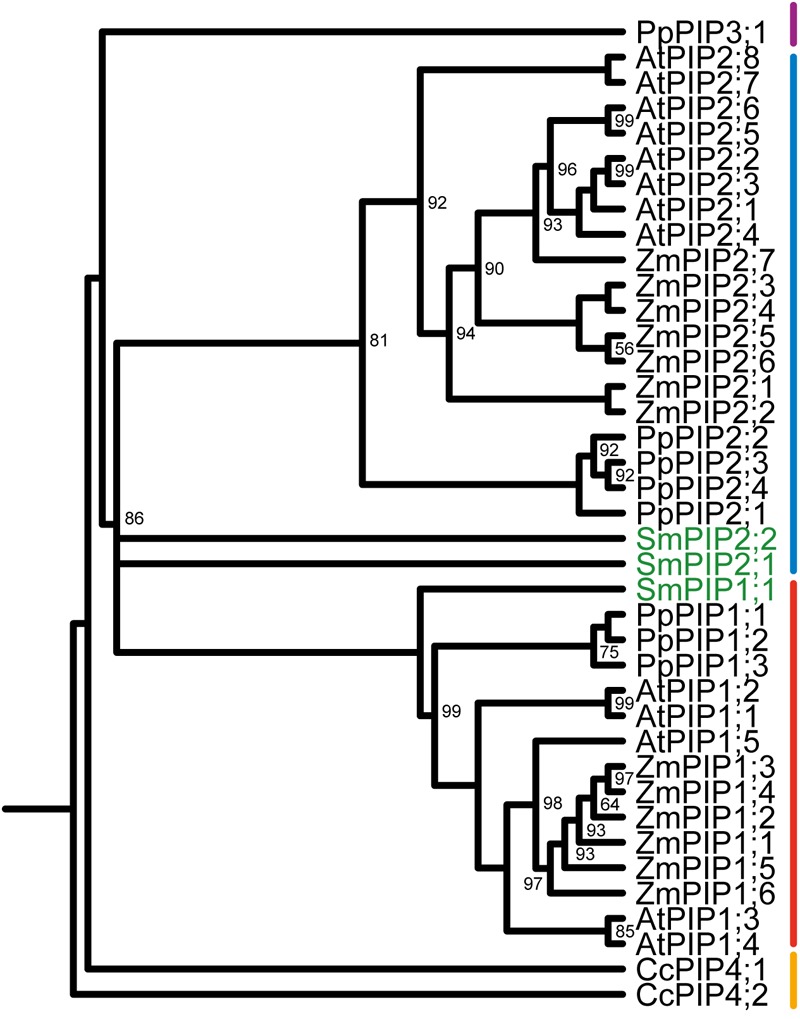
Phylogeny of plasma membrane intrinsic proteins (PIPs) of *Selaginella moellendorffii*. Phylogenetic tree derived from all PIP amino acid sequences of *S. moellendorffii* (Sm), *Zea mays* (Zm), *Arabidopsis thaliana* (At), *Coccomyxa* sp. (Cc), and *Physcomitrella patens* (Pp) using Bayesian phylogenetic inference. Numbers next to branches indicate the percentage of node support for each branch. Only node support percentages <100 are shown. SmPIPs are highlighted in green. The clustering of PIPs into PIP1-, PIP2-, PIP3-, and algae PIP4-subgroups is indicated with a red, blue, purple, or orange line, respectively.

The phylogenetic tree of PIPs from *S. moellendorffii, Arabidopsis thaliana*, and *Zea mays* shows a defined PIP1 and PIP2 clade with strong node supports (**Figure 1**). One *S. moellendorffii* sequence (SmPIP1;1) clusters with the PIP1 group and two sequences (SmPIP2;1 and SmPIP2;2) with the PIP2 group. This is consisting with previous analysis (Anderberg et al., 2012) suggesting that all PIPs of this lycophyte belong to the PIP1 or PIP2 clusters, also occurring in higher plants. This is in contrast to evolutionary more ancient species and taxa such as *Physcomitrella patens*, a moss, or the green algae *Coccomyxa* which possess additional PIP subgroups which have been classified as PIP3 and PIP4 isoforms, respectively (Anderberg et al., 2011, 2012).

SmPIP1;1, SmPIP2;1, and SmPIP2;2 include the two highly conserved NPA motifs, which are a hallmark of AQPs. Their ar/R filters consist of F/H/T(S)/R residues, which are typical for the PIP subfamily and are most likely permeable to water. In addition, several other PIP sequence features are found in SmPIPs (Supplementary File 2), arguing for an early evolution of this plant AQP subfamily. SmPIP2;1 possesses a PIP2-typical ER-exit diacidic motif (DxE) in the N-terminal region. The PIPs conserved histidine in loop D, responsible for Ca^2+^ binding and pH gating, and the phosphorylation site in loop B is also conserved in all three SmPIPs. Interestingly, SmPIP2;2 has some atypical PIP2 sequence features which might have an impact on its subcellular localization. The N-terminus possesses an unconventional diacidic motif (ESE), which has not been studied for its functionality in ER-export, yet. Transmembrane domain 3 of PIP2s encodes a “LxxxA” motif which has been identified to be essential for leaving the ER through the anterograde pathway (Chevalier et al., 2014). Instead, SmPIP2;2 possesses a “FxxxM” motif which is identical to the one in ZmPIP1;2 which has been demonstrated to cause ER retention, also when inserted into a PIP2 protein (Chevalier et al., 2014).

### SmPIP1;1 and SmPIP2;1 Possess Differential Water Channel Transport Activities When Being Heterologously Expressed in *Xenopus* Oocytes

**FIGURE 2:**
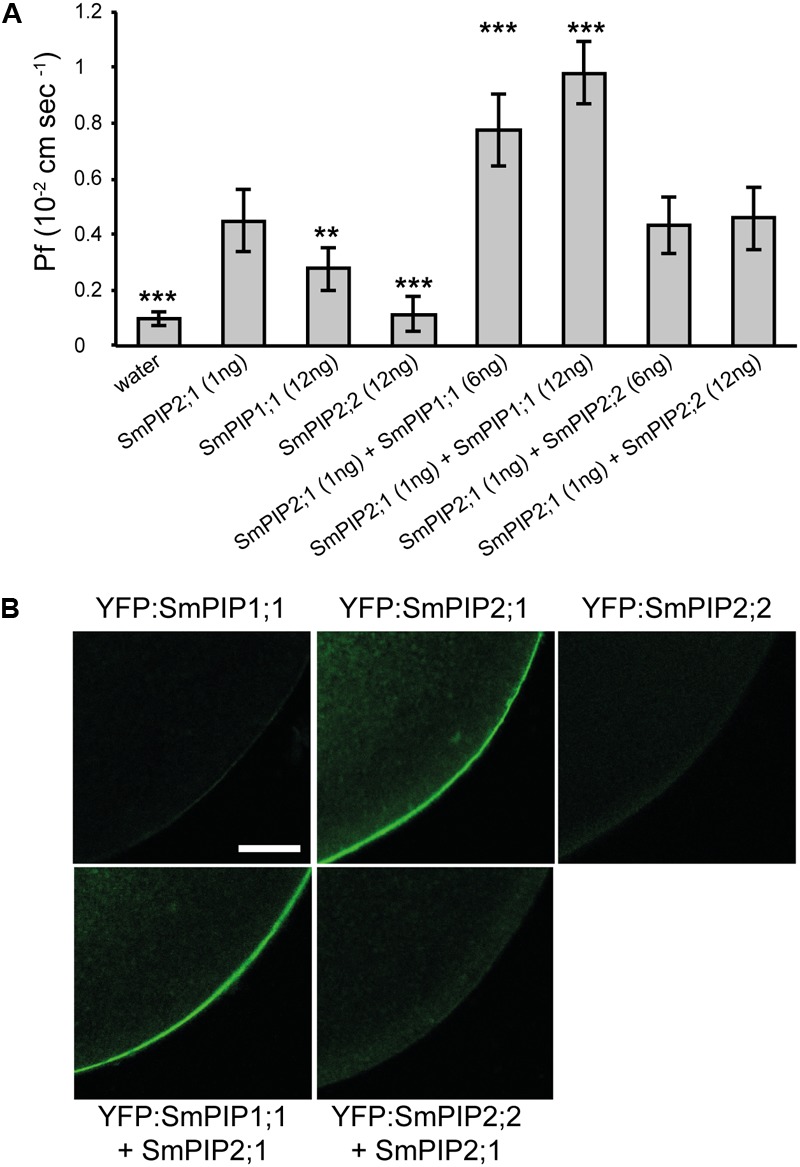
Water permeability coefficient (P_f_) measurements and confocal microscopic images of *Xenopus* oocytes expressing SmPIPs. **(A)** P_f_ values for *Xenopus* oocytes injected with water or expressing SmPIP1;1, SmPIP2;1, or SmPIP2;2, or co-expressing SmPIP1;1 and SmPIP2;1, or SmPIP2;1 and SmPIP2;2. In total, 1 ng of *SmPIP2;1*, or 6 or 12 ng of *SmPIP1;1* or *SmPIP2;2* cRNA was injected. The results are expressed as the mean ± SD (water injected oocytes: *n* ≥ 10, SmPIP injected oocytes: *n* ≥ 9). Significance was calculated using *t*-test. Asterisks mark significant differences to the expression of SmPIP2;1 (^∗∗∗^*p* < 0.001, ^∗∗^*p* < 0.01). The experiment was repeated twice with consistent results. **(B)** Confocal microscopic images of fixed oocytes showing the localization of YFP-tagged SmPIP proteins. Oocytes were injected with 2 ng of *YFP:SmPIP2;1* cRNA or 25 ng of *YFP:SmPIP1;1, YFP:SmPIP2;2*, single- or co-injected with 1 ng *SmPIP2;1* cRNA, then observed 3 days after injection. Representative images are shown.

To quantify the water channel activity of SmPIPs, the membrane osmotic water P_f_ was determined in oocytes injected with cRNA encoding the three isoforms. SmPIP2;1 significantly increased the water permeability of oocytes even if only 1 ng of cRNA was injected (**Figure 2A**). When 2 ng of SmPIP2;1 cRNA was injected the P_f_ raised to 0.944 ± 0.146 × 10^-4^ m/s (data not shown) suggesting a very high water channel activity of SmPIP2;1. The P_f_ values of oocytes expressing SmPIP2;2 and SmPIP1;1 did not significantly differ from water-injected oocytes (negative control), even if a small increase in P_f_ was observed after injecting 12 ng of SmPIP1;1 RNA (**Figure 2A**). To determine whether the absence of P_f_ increase of oocytes expressing singly SmPIP1;1 or SmPIP2;2 was due to a lack of water channel activity or to an absence of the proteins in the plasma membrane, we performed a localization analysis of GFP-tagged SmPIPs. While oocytes injected with 2 ng *GFP:SmPIP2;1* cRNA displayed a clear GFP signal in the oocyte periphery corresponding to the plasma membrane, injection of 25 ng *GFP:SmPIP1;1* or *GFP:SmPIP2;2* cRNA resulted in only a very weak GFP signal in the plasma membrane (**Figure 2B**).

To determine whether non-functional SmPIP1;1 and SmPIP2;2 physically interact with functional SmPIP2;1 leading to a synergistic effect on the membrane permeability, the P_f_ was measured in oocytes co-injected with cRNAs encoding either of the two combinations. When 1 ng of *SmPIP2;1* cRNA was co-injected with increasing amounts of *SmPIP1;1* cRNA (6 or 12 ng), which, alone, had no significant effect on the P_f_, a significant increase in P_f_ was measured compared with oocytes expressing SmPIP2;1 alone (**Figure 2A**). This P_f_ increase was dependent on the amount of injected *SmPIP1;1* cRNA. When 1 ng of *SmPIP2;1* cRNA was co-injected with increasing amounts of *SmPIP2;2* cRNA (6 or 12 ng), no significant increase in the P_f_ was measured compared with oocytes expressing SmPIP2;1 alone (**Figure 2A**). Interestingly, co-injection of 1 ng *SmPIP2;1* with 25 ng *GFP:SmPIP1;1* resulted in a clear plasma membrane GFP:SmPIP1;1 signal while this was not the case for GFP:SmPIP2;2 when co-expressed with SmPIP2;1 (**Figure 2B**). These results are in agreement with the water transport assays and suggest that, when expressed alone, only SmPIP2;1 reaches the oocyte plasma membrane where it facilitates water diffusion, while SmPIP1;1 needs to interact with SmPIP2;1 to be stabilized, reach the plasma membrane, and act as a water channel. This is not the case for SmPIP2;2 that remains in low amount in internal membranes regardless of whether it is expressed alone or co-expressed with SmPIP2;1.

### Co-expression of SmPIP2;1 With SmPIP1;1 or SmPIP2;2 in Yeast Results in the Re-localization of SmPIP1;1 to the Plasma Membrane

**FIGURE 3:**
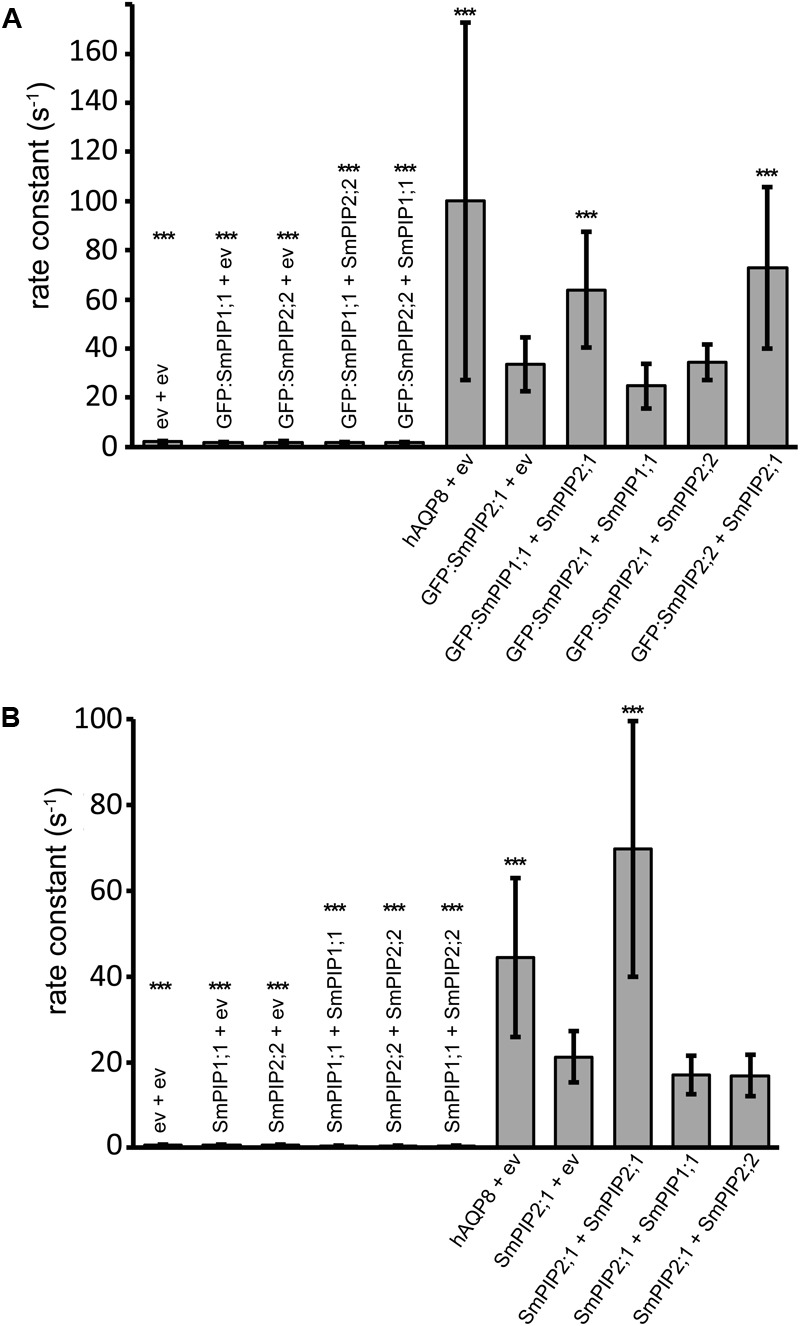
SmPIP-mediated osmotic water transport in yeast analyzed by stopped-flow spectrometry. SmPIP water transport capacity was determined using spheroplasts expressing GFP-tagged **(A)** or non-tagged **(B)** SmPIPs. Spheroplasts were prepared from the wild-type *S. cerevisiae* BY4741 yeast strain transformed with *hAQP8* or empty vectors as controls or with indicated individual SmPIPs or SmPIP combinations. Spheroplasts were suspended in a 1.8 M sorbitol-based buffer at an OD_600_ of 1.5 and mixed in a fast kinetics instrument with an equal volume of a 1.2 M sorbitol-based buffer. Swelling kinetics were recorded by measuring the scattered light intensity over a time period of 6–8 s. Based on the swelling kinetics within the first second of swelling, rate constants were calculated for all trace recordings. To this aim traces were fitted to a one- or two-phase decay equation to obtain the best fit using GraphPad Prism6. Rate constant values for the fitted curves were determined and are displayed in **(A)** and **(B)**. Gray bar charts represent mean values of rate constants ± SD (*n* = 22–28). Significance was calculated using *t*-test. Asterisks mark significant differences (^∗∗∗^*p* < 0.001) to the expression of GFP:SmPIP2;1 + ev in **(A)** or to SmPIP2;1 + ev in **(B)**.

To confirm these results in an independent experimental system, we expressed the three SmPIPs either singly or in combinations in BY4741 yeast cells, and tested their water transport capacity by subjecting yeast spheroplasts to a stopped-flow spectrometric analysis. Trace records of scattered light were monitored and rate constants of the decrease of scattered light intensities (swelling of spheroplasts due to water uptake), which is proportional to water permeability coefficients, were calculated (Kozono et al., 2003; Calamita et al., 2005). Yeast cells expressing GFP:SmPIP1;1 or GFP:SmPIP2;2 had swelling kinetics similar to empty vector negative control cells (**Figures 3A,B**). Human AQP8, a highly water permeable AQP, was used as a positive control (**Figure 3**). Expression of GFP:SmPIP2;1 (**Figure 3A**) or SmPIP2;1 (**Figure 3B**) resulted in a strong increase in water uptake illustrated by a large rate constant value similar to the positive control.

To test the potential synergistic effects of non-functional SmPIP1;1 and SmPIP2;2 on functional SmPIP2;1 in yeast, we co-expressed the three non-tagged SmPIPs with non-tagged SmPIPs (**Figure 3B**) or with GFP-tagged SmPIPs (**Figure 3A**) in all possible combinations and repeated the stopped-flow spectrometric analysis. Only the yeast cells expressing GFP:SmPIP2;1 or SmPIP2;1 alone, or in combinations with SmPIP1;1 or SmPIP2;2, resulted in a swelling of spheroplasts, while the co-expression of SmPIP2;2 with SmPIP1;1 fused or not with the GFP did not increase the spheroplast permeability and showed a similar rate constant as the negative control (**Figure 3**). These data did not allow us to conclude about any synergistic effect on the membrane permeability after the co-expression of SmPIP2;1 with SmPIP1;1 or SmPIP2;2, but demonstrate that co-expression of SmPIP1;1 and SmPIP2;2 did not increase the membrane permeability.

**FIGURE 4:**
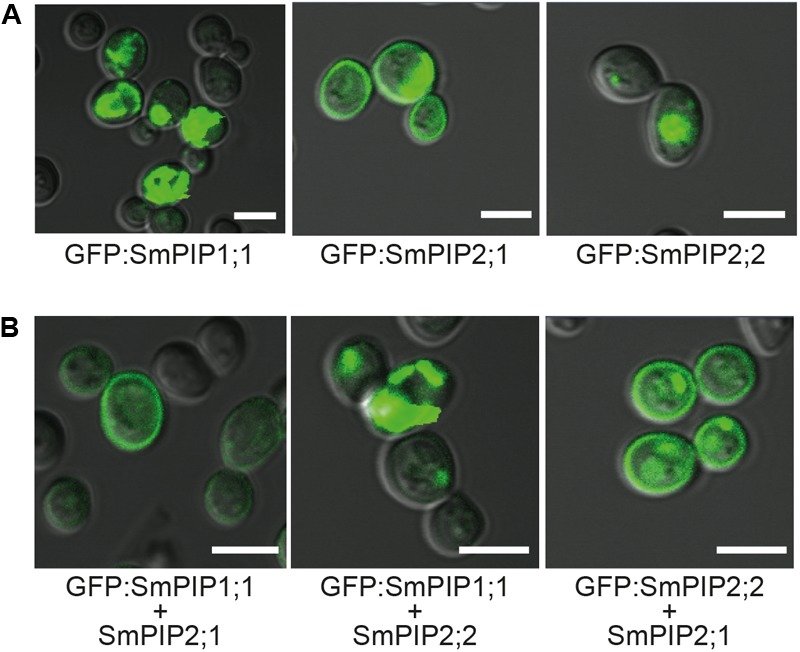
Localization of N-terminally GFP-tagged SmPIP proteins in yeast cells. Wild-type *S. cerevisiae* cells (BY4741) of the exponential growth phase (OD_600_ = 1–1.3) expressing GFP:SmPIP1;1 or GFP:SmPIP2;1 or GFP:SmPIP2;2 **(A)** or co-expressing GFP:SmPIP1;1 and SmPIP2;1, GFP:SmPIP1;1 and SmPIP2;2, or GFP:SmPIP2;2 and SmPIP2;1 **(B)** were examined by confocal microscopy. An overlay of the GFP channel and the Nomarski optical transmission is displayed. Scale bars = 5 μm.

We then investigated the amount of protein and localization of SmPIPs (co-)expressed in yeast, thanks to the presence of the fused GFP. As shown in **Figure 4A**, yeast expressing GFP:SmPIP2;1 displayed a clear GFP signal around the cells corresponding to the plasma membrane, in addition to signals in internal structures, while GFP:SmPIP1;1 and GFP:SmPIP2;2 expressing yeast cells showed fluorescent signals predominantly in internal structures, but not in the plasma membrane. These localization data are similar to the results obtained in *Xenopus* oocytes, even if, in the latter system, the intensity of the internal signal was much lower for SmPIP1;1 and SmPIP2;1, suggesting that the intracellular retained proteins were more rapidly degraded in oocytes. To investigate whether SmPIP2;1 controls the amount and/or stability of SmPIP1;1 in the yeast plasma membrane as observed in oocytes, we assayed the localization of GFP:SmPIP1;1 in cells co-expressing SmPIP2;1. As shown in **Figure 4B**, a bright and sharp GFP signal was detected in the plasma membrane, similar to that seen with GFP:SmPIP2;1 expressed alone (**Figure 4A**), demonstrating that SmPIP1;1 is localized in the yeast plasma membrane when co-expressed with SmPIP2;1. Interestingly, a similar re-localization from internal structures to the plasma membrane was observed for GFP:SmPIP2;2 when being co-expressed with SmPIP2;1, suggesting that, in yeast, SmPIP2;1 is able to interact with and traffic SmPIP2;2 to the plasma membrane. This was not observed in *Xenopus* oocytes. Finally, when co-expressed with SmPIP2;2, GFP:SmPIP1;1 remained in internal membrane, suggesting that both proteins did not interact or their interaction did not lead to a SmPIP1;1 relocalization to the plasma membrane.

### SmPIP2;1 Homotetramer or as Heterotetramer With SmPIP1;1 or SmPIP2;2 Increases the Sensitivity of Yeast Cells to Hydrogen Peroxide

We demonstrated that SmPIP2;1 homotetramers as well as SmPIP2;1/SmPIP2;2 and SmPIP2;1/SmPIP1;1 heterotetramers (interaction leading to SmPIP1;1 or SmPIP2;2 re-localization) are functionally expressed in the yeast plasma membrane. To determine the substrate specificity of SmPIPs for other solutes than water and to unravel potential functional and specificity changes upon heteromerization, the three SmPIPs were singly and co-expressed in all possible combinations in different yeast strains capable of uncovering permeability to hydrogen peroxide, boric acid, ammonia, and urea, which are other putative PIP substrates.

**FIGURE 5:**
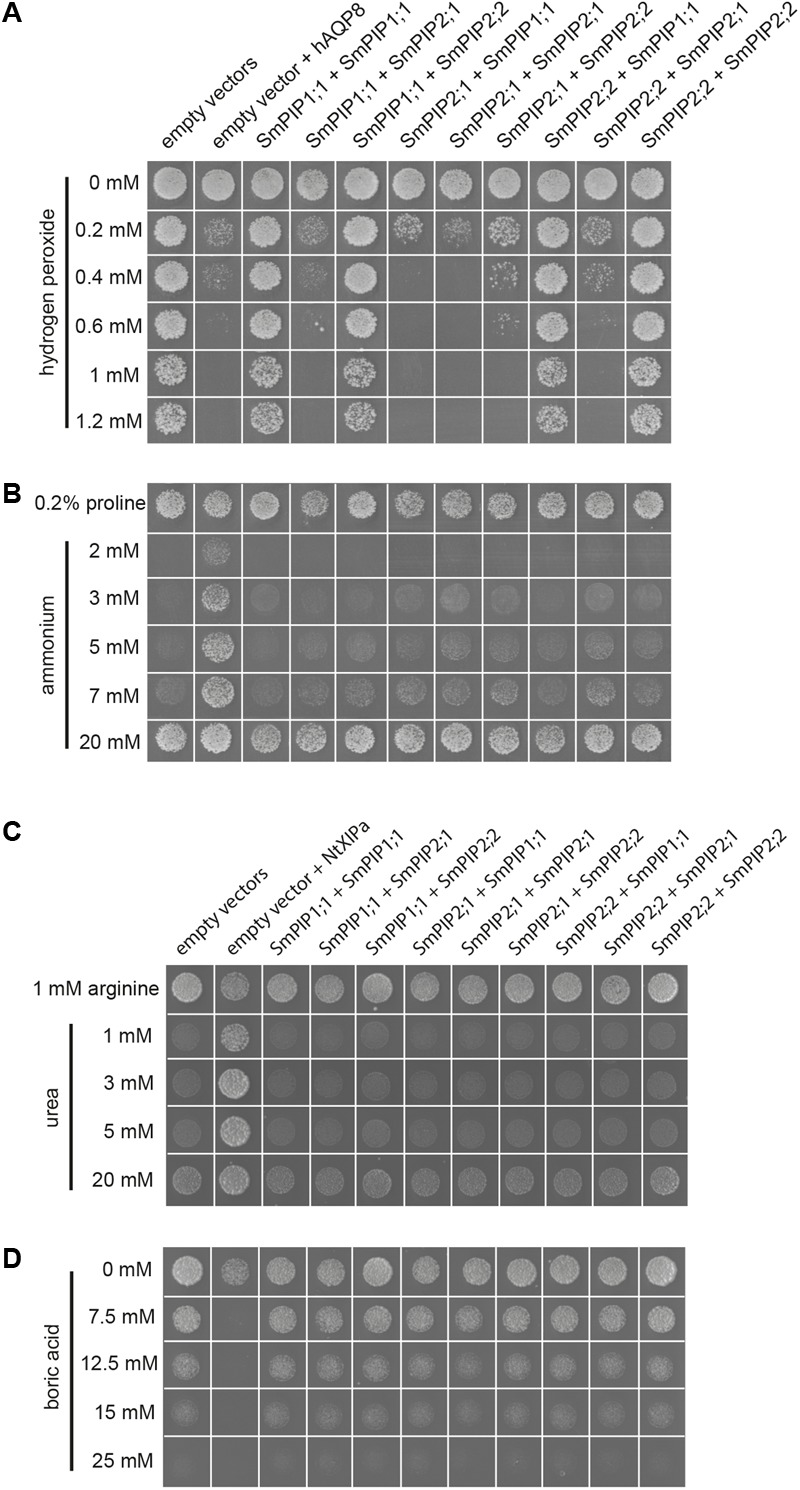
Substrate specificity studies of SmPIPs in yeast by growth complementation- or toxicity growth assay. Hydrogen peroxide **(A)** and boric acid **(D)** toxicity growth assays and ammonium **(B)** and urea **(C)** complementation assays in the *Δmep1-3* **(A+B)** and *Δdur3* **(C+D)** mutant yeast strains expressing SmPIP isoforms. Cultures of mutant yeast cells co-transformed with the indicated combinations of empty vectors (pYeDP60u-ura or pYeDP60u-leu), pYeDP60u-leu, and pYeDP60u-ura carrying *hAQP8*, pYeDP60u-leu and pYeDP60u-ura carrying *NtXIP1;1α*, or pYeDP60u-ura and pYeDP60u-leu carrying the indicated *SmPIP* cDNA were diluted in sterile distilled water to an OD_600_ of 0.01 and spotted on medium containing the indicated concentrations of hydrogen peroxide **(A)**, boric acid **(D)**, ammonium **(B)**, proline **(B)**, arginine **(C)**, or urea **(C)**. The growth behavior and survival rates of the different transformants were recorded after 7–10 days at 30°C and were shown for the yeasts spotted at an OD_600_ of 0.01. All yeast growth assays were performed at least twice, with consistent results. Displayed images in **(A–D)** represent groups of sub-images assembled from different growth plates and conditions. Each “yeast growth spot” represents a sub-image within the image. This procedure does not alter any information and represents a usual presentation practice of yeast growth and complementation assays.

Aquaporins facilitate the diffusion of the essential signaling molecule H_2_O_2_ across biological membranes (Bienert et al., 2007; Dynowski et al., 2008b; Bienert and Chaumont, 2014). This is also the case for AtPIP1;4 and AtPIP2;1 *in planta*, in which this channel specificity contributes to pathogen resistance and stomata regulation, respectively (Tian et al., 2016; Rodrigues et al., 2017). To test whether SmPIPs can mediate the diffusion of H_2_O_2_, the *Δmep1-3* yeast-mutant strain was transformed with vectors expressing the different SmPIP isoforms alone or in combination, and hAQP8 as a positive control, and exposed to increasing amounts of H_2_O_2_ in the external growth medium. The addition of 1.2 mM H_2_O_2_ did not decrease the growth of cells containing the empty vector, or cells expressing SmPIP1;1 or SmPIP2;2 alone or in combination (**Figure 5A**). However, expression of SmPIP2;1 homotetramers or heterotetramers, in combination with SmPIP1;1 or SmPIP2;2, markedly reduced cell survival on medium containing 0.2–0.4 mM H_2_O_2_ to a similar extent as that of the positive control hAQP8. These data were confirmed in multiple repetitions and were not dependent on the plasmid expressing the SmPIP isoforms (**Figure 5A**) nor on the used yeast strains (data not shown).

To determine whether SmPIPs facilitate the diffusion of ammonia across membranes, a yeast complementation assay was performed. The yeast mutant *Δmep1-3*, which carries deletions in all three yeast *mep* transporter genes, is unable to grow on medium containing 5 mM or lower concentrations of ammonium as the sole nitrogen source due to missing ammonium uptake capacity, but can grow on other nitrogen sources such as the amino acid proline (control medium). While the expression of the positive control, hAQP8, an aquaammoniaporin (Jahn et al., 2004; Saparov et al., 2007) clearly complemented the mutant phenotype and rescued the cell growth below 5 mM ammonium in the growth medium, none of the cells being transformed with *SmPIP1;1, SmPIP2;1, SmPIP2;2*, or combinations thereof grew under these conditions (**Figure 5B**).

To determine whether SmPIPs facilitate the membrane diffusion of urea, the yeast mutant *Δdur3*, which possesses a deletion in the DUR3 urea transporter and is incapable to grow on medium containing ≤ 5 mM urea as the sole nitrogen source but on other nitrogen sources such as arginine (Liu et al., 2003) was used. While the expression of NtXIP1;1α, which was used as a positive control (Bienert et al., 2011), clearly complemented the mutant phenotype and rescued cell growth below 5 mM urea in the growth medium, none of the cells being transformed with *SmPIP1;1, SmPIP2;1, SmPIP2;2*, or combinations thereof grew under these conditions (**Figure 5C**).

The DUR3 transporter has also been demonstrated to be a pathway for boric acid influx into yeast cells (Nozawa et al., 2006). Therefore, the *Δdur3* mutant is more resistant to boric acid toxicity than wild-type yeast (Nozawa et al., 2006) and can be used in a toxicity growth assay to identify transport proteins with a permeability to boric acid. As being permeable to boric acid, NtXIP1;1α was used as a positive control in this assay (Bienert et al., 2011). While the expression of NtXIP1;1α clearly increased the sensitivity of the yeast cells already at 7.5 mM boric acid, none of the cells being transformed with *SmPIP1;1, SmPIP2;1, SmPIP2;2*, or combinations thereof showed a decreased survival rate or growth ability compared to the empty vector negative control up to 15 mM boric acid (**Figure 5D**). None of the yeast transformants was able to significantly grow at 25 mM demonstrating the toxic effect of boric acid on yeast cells.

### SmPIP1;1 and SmPIP2;1 Interact *in Planta* to Re-localize SmPIP1;1 to the Plasma Membrane

**FIGURE 6:**
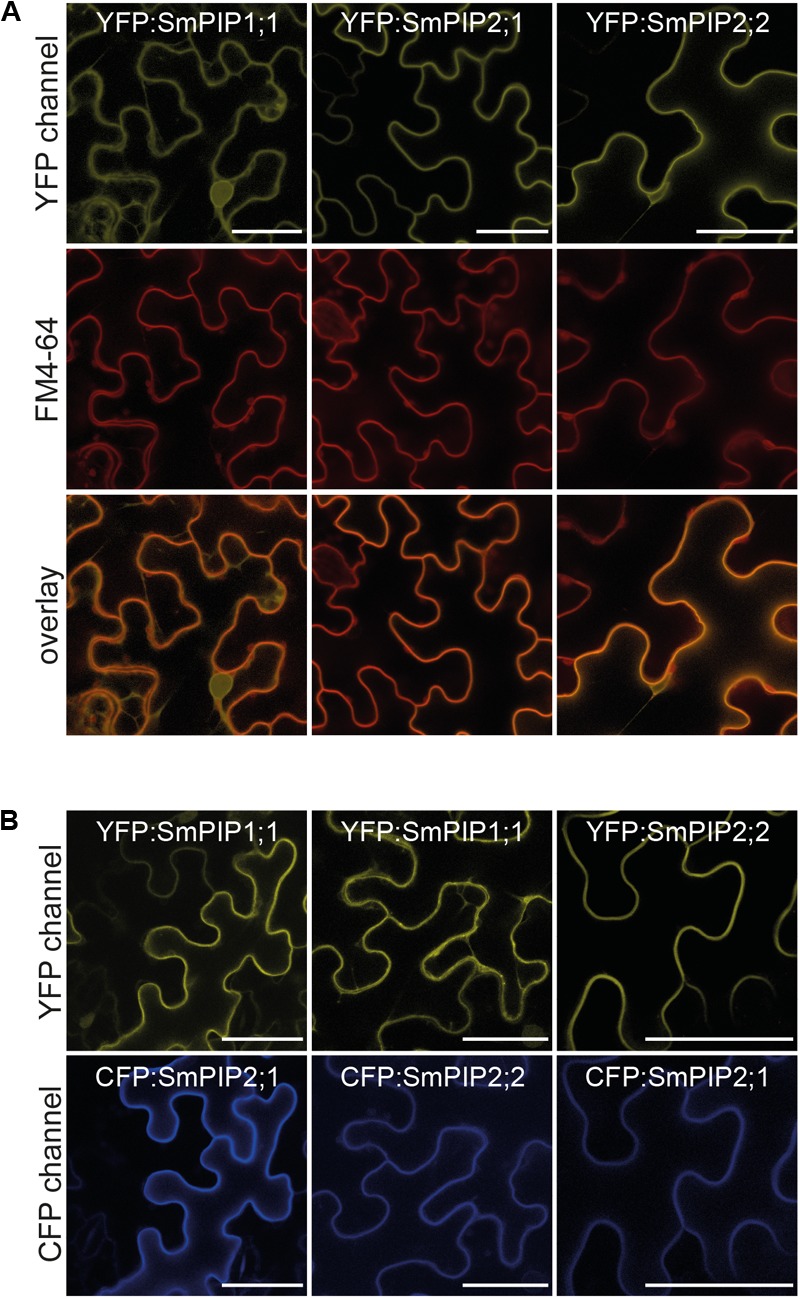
Subcellular localization of SmPIPs transiently expressed in *Nicotiana benthamiana* epidermis cells. **(A)** Abaxial tobacco epidermis cells infiltrated with the plasma membrane marker FM4-64 transiently expressing mYFP:SmPIP1;1, mYFP:SmPIP2;1, or mYFP:SmPIP2;2. Scale bars = 40 μm. **(B)** Abaxial tobacco epidermis cells co-expressing mYFP:SmPIP1;1 and mCFP:SmPIP2;1, mYFP:SmPIP1;1 and mCFP:SmPIP2;2, or mYFP:SmPIP2;2 and mCFP:SmPIP2;1. Scale bars = 50 μm. Representative images are displayed.

Having demonstrated that SmPIP1;1 and SmPIP2;1 interact in *Xenopus* oocytes and yeast to allow the trafficking of the former to the plasma membrane, we analyzed whether this regulatory process also occurs *in planta*. Therefore, SmPIP1;1, SmPIP2;1, and SmPIP2;2 were fused to the C-terminus of mCFP and/or mYFP. The subcellular localization of each SmPIP fusion protein was determined in *A. tumefaciens*-mediated transiently transformed tobacco abaxial epidermis leaf cells by confocal microscopy (**Figure 6**). Cells transiently expressing mYFP:SmPIP2;1 displayed sharp YFP signals at the plasma membrane (**Figure 6A**), as confirmed by co-localization with the plasma membrane marker FM4-64 (**Figure 6A**, red fluorescence). mYFP:SmPIP2;2-derived fluorescence was detected in the plasma membrane but also in punctate structures and in intracellular structures probably corresponding to the ER (**Figure 6A**). mYFP:SmPIP1;1 was found in the plasma membrane but dominantly in punctuated and diffuse internal structures within the cytosol. Based on the fluorescent signal pattern and co-localization with the plasma membrane marker FM4-64, SmPIP1;1 might be predominantly localized to the ER. Upon co-expression of mYFP:SmPIP1;1 and mCFP:SmPIP2;1, or mYFP:SmPIP2;2 and mCFP:SmPIP2;1, mYFP:SmPIP1;1 and mYFP:SmPIP2;2 were found in the cell plasma membrane together with mCFP:SmPIP2;1 (**Figure 6B**). However, co-expression of mYFP-SmPIP1;1 and mCFP-SmPIP2;2 did not result in a sole localization of mYPF-SmPIP1;1 in the plasma membrane but was partly retained in the ER.

## Discussion

*Selaginella moellendorffii* is a model clubmoss plant and has a well-characterized genomic sequence. This group of land plants possesses real roots and a vascular tissue while true leaves are still absent. The water homeostasis of this life-form probably demands an already complex regulation. AQPs, and in particular PIPs, have been demonstrated to fulfill such hydraulic regulatory functions. The genome of *S. moellendorffii* codes for three PIPs which, in all likelihood, contribute to the regulation of the plant water relations through the control of cell membrane water fluxes. Therefore, it is interesting to characterize SmPIP1;1, SmPIP2;1, and SmPIP2;2 in detail. Available transcriptome data sets show that SmPIP1;1 and SmPIP2;1 are expressed together in the meristematic zone of *Selaginella* roots (Huang and Schiefelbein, 2015). Moreover, elucidating PIPs from a taxon, which formed early in the evolution of land plants, is highly interesting from a PIP functional evolutionary point of view. So far, experimental studies on PIPs focus on isoforms of angiosperms, a rather recently developed clade of land plants. The original functional properties and selectivities of plant PIPs are almost unknown to date. Liénard et al. (2008) functionally studied PpPIP2;1, PpPIP2;2, and PpPIP2;3 which are expressed in the gametophore of *P. patens*. Direct water permeability measurements and physiological analyses of moss mutants being knocked-out for these individual PIPs suggest that PpPIP2;1 and PpPIP2;2 facilitate the uptake of water into moss cells while PpPIP2;3 seems to be impermeable to water (Liénard et al., 2008). Interactions between these PpPIPs have not been investigated. In this study, we molecularly and functionally characterized all PIP isoforms of *S. moellendorffii* in terms of water transport, substrate specificity, and the ability to form heteromeric functional units.

SmPIPs can phylogenetically clearly be assigned to the PIP1 and PIP2 subgroup which occur in all higher plants. Soto et al. (2012) suggested that PIPs can be divided into three clusters. Based on the phylogenetic organization, the authors subdivided PIP2s into two clusters with specific conserved motifs for each subfamily and each cluster. Applying the method for classification on SmPIPs in this study indicated that *Selaginella* possesses one PIP isoform belonging to each of the three clusters (SmPIP1;1 = PIP-like cluster I; SmPIP2;1 = PIP-like cluster III; SmPIP2;2 = PIP-like cluster II).

It has been established for certain PIP1s and PIP2s of higher plants that they have different water transport abilities when expressed in the heterologous oocyte and yeast expression systems. While PIP2 isoforms reach the plasma membrane as functional tetramers to facilitate the diffusion of water along a concentration gradient, most PIP1s do not (reviewed in Jozefkowicz et al., 2017). However, upon co-expression PIP1s and PIP2s functionally interact to allow PIP1s to reach the plasma membrane and modify their transport activity/selectivity (reviewed in Jozefkowicz et al., 2017). So far, evidence is lacking whether PIP1/PIP2 interaction is incidentally and solely based on the fact that these proteins are sequence-wise highly similar (about 80% identity) and can therefore assemble in heterotetramers (tetramers being the typical structure for AQPs), or whether PIP1/PIP2 interaction is biologically controlled and employed to regulate transport processes. It is unlikely that plants would conserve two PIP subgroups which are putatively identical in function for redundancy reasons. This argues for the fact that PIP1s and PIP2s either have clearly distinct transport functions, for which no unequivocal evidence exists yet, or that upon heterotetramerization PIP1/PIP2 pairs gain unique properties of high physiological relevance which cannot be accomplished by a homotetramer of only one group of PIPs.

So far, studies elucidating the role of PIP1/PIP2 pairs and the consequences of PIP1/PIP2 interaction on functionality, such as the water transport, have mainly focused on the experimental oocyte system. A few studies have been performed in yeast. For instance, native ZmPIP1;2 and ZmPIP2;5 have been co-expressed in yeast to investigate consequences on H_2_O_2_ but not on water transport (Bienert et al., 2014). Artificial heterotetramers with a defined proportion of NtAQP1 to NtPIP2;1 monomers have been studied with the aim to quantify water versus CO_2_ permeability in dependence of the PIP1/PIP2 isoform proportion (Otto et al., 2010).

The present study allows a preliminary rating whether the yeast expression system is experimentally suitable to elucidate functional consequences of PIP heteromerization such as the synergistic water transport effect or not. Although the oocyte water transport assay provided clear evidence for a synergistic water transport effect of the SmPIP1;1/SmPIP2;1 pair, no increase in water flux rate has been observed following co-expression of SmPIP1;1 and SmPIP2;1 compared to SmPIP2;1 expression alone in the yeast system. Similarly, no elevated rate constant has been observed upon co-expression of SmPIP1;1 and GFP:SmPIP2;1 compared to GFP:SmPIP2;1 expression alone in yeasts. These results strongly suggest that a synergistic effect on water membrane permeability is absent in the yeast system. A missing synergistic effect on water transport upon co-expression of the SmPIP2;1/SmPIP1;1 pair in yeasts compared to oocytes might be explained by either a differential post-translational regulation of PIP channel activity or a different phospholipid membrane environment in these two biological systems. Recent studies have suggested that the molecular composition of lipid bilayers can modify the spatial organization of transmembrane helices of membrane proteins through direct chemical interactions (Opekarová and Tanner, 2003; Lee, 2011). For instance phospholipid-mediated alteration in α-helix packing may influence protein stability, protein trafficking efficiency to a certain cellular compartment, or the membrane protein activity itself (Opekarová and Tanner, 2003; Lee, 2011). Interestingly, Otto et al. (2010) observed also no synergistic effect on yeast water membrane permeability when artificial NtAQP1/NtPIP2;1 heterotetramer or homotetramer combinations have been evaluated by stopped-flow analysis. Therefore, future analysis will have to demonstrate whether an observed missing synergistic effect in yeast is a peculiarity of certain PIP pairs (such as SmPIP2;1/SmPIP1,1 or NtPIP2;1/NtAQP1) or whether this is a general difference between the oocyte and yeast expression system. In the latter case, the molecular reason for this, e.g., the impact of different phospholipid environments on different plant PIP isoforms, remains to be identified in further studies.

Co-expression of GFP:SmPIP1;1 and SmPIP2;1 resulted in higher rate constants compared to GFP:SmPIP2;1 expression alone or to co-expression of GFP:SmPIP2;1 and SmPIP1;1 (**Figure 3A**). Similarly, co-expression of GFP:SmPIP2;2 and SmPIP2;1 resulted in higher rate constants compared to GFP:SmPIP2;1 expression alone or to co-expression of GFP:SmPIP2;1 and SmPIP2;2 (**Figure 3A**). This suggests that either less translational products of GFP-tagged compared to non-tagged SmPIP2;1 exist or that GFP is influencing the PIP targeting to the plasma membrane or its transport ability therein. Decreased water transport rates of GFP-tagged SmPIP2;1 compared to non-tagged SmPIP2;1/PIP pairs and the significantly increased rate constant of spheroplasts co-expressing SmPIP2;1 and SmPIP2;1 on two vectors compared to SmPIP2;1/empty vector expression (**Figure 3B**) suggest that the GFP-tag influences the amount of functional PIP2 protein in the yeast plasma membrane. One critical difference between the yeast and the oocyte system is that the expression level of the gene-of-interest can be more easily controlled in oocytes by adjusting the amount of cRNA to be injected. Even though the protein translation in different oocytes will vary to a certain degree upon cRNA injection, the genetic material to be translated is fine-controlled. In contrast, the usage of multicopy plasmids, as being used in this study, will result in yeast cells with different plasmid numbers, subsequent different transcript amounts, and finally different amounts of protein being translated and inserted into the plasma membrane. Therefore, the absolute quantification of transport rates between different transformants and even batches of the same transformant might have to be taken with care if the absolute protein amount in the plasma membrane is unknown. The quantification of the latter is also far from being trivial as heterologously expressed membrane proteins normally end-up in internal yeast membranes to a significant extend rather than in the plasma membrane which is, in general, in contrast to the oocyte system. On the other hand, translational differences between transformants are assumed to be eliminated and averaged out as each stopped-flow measurement is (i) performed with a sample consisting of a heterogeneous batch of a large spheroplast number, (ii) repeated several times with heterogeneous batches of the same transformation event, and (iii) repeated with independent transformants.

The confocal microscopic analyses demonstrated that the yeast system is suitable to visualize PIP1/PIP2 interactions and to determine the subcellular localization of PIP AQPs allowing to anticipate their subcellular localization in plants. This observation is in accordance with previous studies (Otto et al., 2010; Bienert et al., 2014). One disadvantage of the yeast system is that not all cells display a uniform gene expression and, subsequent, differentially intense fluorescent signals in the different membrane systems.

SmPIP2;2 showed sequence characteristics which are typical for PIP2 proteins and clusters phylogenetically with the PIP2 subfamily. However, SmPIP2;2 shares few characteristics with PIP1 AQPs. These are mostly in relation to protein trafficking and subcellular localization features, such as an uncommon diacidic motif (ESE) (Zelazny et al., 2009) and a missing typical PIP2-type “LxxxA” motif which was shown to be important for ER exit (Chevalier et al., 2014). These sequence peculiarities may explain why SmPIP2;2 resides in intracellular structures and does not induce water transport activity both in oocytes and yeasts. Interestingly, PpPIP2;3 from *P. patens* another PIP2 from non-seed plants without a diacidic motif has also been shown to be incapable to facilitate transmembrane water transport (Liénard et al., 2008). In the yeast system, SmPIP2;1 interacted with SmPIP2;2 to re-localize it to the plasma membrane. This was not observed in the oocyte system.

Based on all obtained information, SmPIP2;2 behavior is more difficult to interpret than that of SmPIP1;1 and/or SmPIP2;1. In the yeast system, GFP-tagged SmPIP2;2 remains intracellular while in transiently transformed tobacco cells SmPIP2;2 is found in both the plasma membrane and internal structures. Possible explanations for the plasma membrane localization would be an interaction with endogenously expressed tobacco PIP2 isoforms which results in the trafficking of SmPIP2;2 to the plasma membrane or a protein modification which occurs *in planta*, but not in yeast or oocyte cells. An alternative explanation for the observed differences of XFP-tagged SmPIP2;2 localizations in the different expression systems may also be, as described-above, the contrasting phospholipid environment prevailing in these different biologic systems.

Plasma membrane intrinsic protein heterotetramerization has been proposed to control transport activity and selectivity. PIP isoforms have been suggested to facilitate the diffusion of glycerol (Biela et al., 1999), urea [Gaspar et al., 2003 (ZmPIP1;5)], boric acid [Dordas et al., 2000 (ZmPIP1;1); Dordas and Brown, 2001; Fitzpatrick and Reid, 2009 (HvPIP1;3 and HvPIP1;4); Kumar et al., 2014 (OsPIP2;4 and OsPIP2;7)], arsenous acid [Mosa et al., 2012 (OsPIP2;4, OsPIP2;6, and OsPIP2;7)], hydrogen peroxide (reviewed in Bienert and Bienert, 2017), and carbon dioxide (reviewed in Uehlein et al., 2017). Recently, a permeability of AtPIP2;1 to Na^+^ ions was detected (Byrt et al., 2017). We investigated whether SmPIPs possess permeability to other substrates than water and whether co-expression of SmPIPs results in novel- or prevented existing substrate fluxes. The yeast toxicity assay demonstrated that the expression of SmPIP2;1 alone or the expression of SmPIP2;1/SmPIP1;1 and SmPIP2;1/SmPIP2;2 pairs did not result in a significantly altered H_2_O_2_ sensitivity of yeasts. H_2_O_2_ permeability of water-permeable PIP2s was previously modeled by molecular dynamic simulations and experimentally verified (reviewed in Bienert and Chaumont, 2014; Bienert and Bienert, 2017). Water and H_2_O_2_ have similar physico-chemical properties with respect to the demands needed to permeate through an AQP (Bienert et al., 2006) and co-permeability for these two substrates has been suggested for water permeable AQPs (Almasalmeh et al., 2014). Our study confirmed this dual substrate selectivity and suggests that H_2_O_2_ permeable PIP AQPs had developed already in non-vascular plants.

A recent study had demonstrated that AtPIP1;4 is of physiological importance in the transduction of externally generated H_2_O_2_ signals across the plasma membrane into the cytosol to activate a signaling cascade which is crucial to counteract pathogen attacks (Tian et al., 2016). The co-expression of SmPIP1;1 and SmPIP2;1 did not increase the sensitivity toward H_2_O_2_ in the external growth medium compared to single expression of SmPIP2;1. Additionally, we could not detect H_2_O_2_ permeability of SmPIP1;1 when being co-expressed with an almost H_2_O_2_ impermeable ZmPIP2;5H199K isoform (data not shown) which is able to traffic PIP1s to the plasma membrane (Bienert et al., 2014). A similar absence of H_2_O_2_ permeability has been observed for ZmPIP1;2 in a heteromer with non-functional ZmPIP2;5s, though the complex being detected in the plasma membrane (Bienert et al., 2014). Either SmPIP1;1 and ZmPIP1;2 are not permeable to H_2_O_2_ (compared to, e.g., AtPIP1;4) or the heterotetrameric conformational PIP1/PIP2 organization prevents H_2_O_2_ permeability through PIP1s. Data showing that heteromerization influences mercury sensitivity and CO_2_ permeability point to the fact that the conformational packing of PIP1 or PIP2 monomers in PIP1/PIP2 heterotetramers is unequal from that in homotetramers. Such structural changes may have consequences on functionality and substrate selectivity (Otto et al., 2010; Bienert et al., 2012). Underlying molecular reasons and the question why specific PIP1s are permeable to H_2_O_2_ and other, sequence-wise very similar, isoforms not, has not been addressed yet. The structural basis and underlying molecular reasons for permeability to H_2_O_2_ in PIP1 and PIP2 homotetramers or the absence in heterotetramers have to be elucidated in future.

The nitrogen metabolism is tightly linked to the water status of the plant and to the activity and expression of AQPs (reviewed in Wang et al., 2016). Nitrogen- and water uptake into plant roots is highly correlated. For instance, nitrate was suggested to be a decisive signaling factor for water fluxes into and along roots (Wang et al., 2016). In *Arabidopsis*, root hydraulic conductivity and PIP expression are controlled by external and internal nitrate concentrations (Li et al., 2016). Physiologically relevant ammonia and urea transport regulation across the tonoplast membrane is suggested to be regulated by aquaammoniaporins, members of the TIP AQP subfamily (Liu et al., 2003; Kirscht et al., 2016). TIP-mediated transport of ammonia and urea into the vacuole allows for nitrogen storage under nitrogen surplus conditions. When plants run short of nitrogen, those nitrogen species can be effluxed out of the vacuole by the same AQPs and therewith re-allocated for the metabolism. Similar nitrogen transport functions of plasma membrane-localized plant AQPs have been speculated (Wang et al., 2016). We tested whether SmPIPs facilitated the diffusion of urea or ammonia. As shown in **Figures 5B,C**, SmPIP homotetramers neither transport these compounds nor is the ability introduced upon heteromerization. Using molecular simulations with the tetrameric plant plasma membrane AQP SoPIP2;1, Dynowski et al. (2008a) showed that ammonia crosses the PIP2 pore less rapidly, while urea conduction was impaired compared to H_2_O conduction. Compared to those of water, the calculated major barriers for ammonia and urea were 4 and 5 kJ/mol higher, respectively. This questions whether PIPs can significantly facilitate the flux of these compounds across native membranes. Accordingly, expression of AtPIP2;1 was not able to mediate sufficient ammonia and urea uptake to enable yeast growth under limited ammonia or urea supply (Dynowski et al., 2008a). Our experimental results strengthen the data of Dynowski et al. (2008a) that homo-tetrameric PIP2 proteins are impermeable to urea and ammonia and extent our knowledge that PIP2 heterotetramerization with PIP1 channels do neither induce permeability to urea nor to ammonia.

Using yeast toxicity growth assays as a read-out, we did not observe any reduction in the survival rate of any of the SmPIP transformants compared to the negative control when being exposed to toxic boric acid concentrations in the growth medium. Our results may suggest that PIP permeability to boric acid, which was described elsewhere (Dordas et al., 2000; Dordas and Brown, 2001; Fitzpatrick and Reid, 2009; Mosa et al., 2016), has developed later during evolution of land plants. However, the physiological relevance of AQP-mediated boric acid transport processes in plants has only been demonstrated for members of the plant NIP AQP subfamily by forward and reverse genetic approaches (reviewed in Pommerrenig et al., 2015). The physiological involvement of PIPs in uptake and translocation of boron in plants remains to be demonstrated. Together, these results suggest that PIPs of *Selaginella* are involved in water and H_2_O_2_ transport processes but not in the transport regulation of urea, ammonia, or boric acid. Permeability to other substrates might be discovered in future.

## Conclusion

Our study shows that the characteristics known for modern PIP1 and PIP2 isoforms of angiosperms in terms of their water transport activity and trafficking emerged already as early as in the evolution of vascular plants. SmPIP1;1 and SmPIP2;1 represent sequence- and functional-wise a classical PIP1/PIP2 pair. The existence of PIP sub-group-specific characteristics and physical PIP1/PIP2 interactions in phylogenetically classified old plant species and their conservation in phylogenetically modern plant species argues for the fact that a strong evolutionary selection pressure was acting on these characteristics and therefore on the complementary functional nature of PIP1 and PIP2 proteins. This further suggests that the heterotetramerization among PIP1 and PIP2 isoforms is conserved and that these functional units are of biological relevance. A co-existence of two distinct PIP groups, just due to isoform redundancy reasons is unlikely. The exact biological role of single PIP1 isoforms remains to be shown and is suggested to be linked to their interaction with specific PIP2s and *vice versa*.

Although concrete evidence for specific physiological functions of PIP1/PIP2 pairs with defined isoform compositions is pending, outstanding indications from literature suggest specific roles in gating, selectivity, pH sensing, or membrane localization. Plant species, such as *S. moellendorffii*, with a small number of PIPs in their genomes have the advantage that one can identify roles of PIP1/PIP2 pairs due to an experimentally manageable amount of combinatory heterotetramerization events. Alternatively, cell types or tissues in which transcription profiles of certain PIP1s and PIP2s are linked are prone to identify the role of the corresponding PIP1/PIP2 functional unit. Specific PIP1–PIP2 pairs might serve as pioneer heterotetramer complexes allowing to understand structural, functional, and regulatory consequences of transport protein oligomerization.

## Author Contributions

GB conceived the research and drafted the manuscript with the input of all authors. All authors approved the final manuscript. TD performed the phylogenetic analyses and subcellular localization analysis in plants and yeast. MB performed the subcellular localization analyses in plants. MB and GB performed the growth, complementation, and water transport assays in yeasts. NR and FC performed the oocyte water transport assays and the localization analyses in oocytes. TD, MB, NR, FC, and GB collected and analyzed the data.

## Conflict of Interest Statement

The authors declare that the research was conducted in the absence of any commercial or financial relationships that could be construed as a potential conflict of interest.
