# Supplementary material for: Heterotetramerization of Plant PIP1 and PIP2 Aquaporins Is an Evolutionary Ancient Feature to Guide PIP1 Plasma Membrane Localization and Function
Source: Front Plant Sci. 2018 Mar 26;9:382. doi: 10.3389/fpls.2018.00382 (PMC5879115; doi:10.3389/fpls.2018.00382)
Supplement: Supplementary file 1 [file Table_1.DOCX]

**Supplementary File 1:** List of primers which have been used in the study. Displayed primers have been used to USER-PCR amplify the cDNAs of the indicated SmPIP aquaporins. PCR products have been cloned in the indicated USER-compatible expression vectors.

| Primer | Tm in °C | Sequenz | Vector |
| --- | --- | --- | --- |
| SmPIP11_Scu_FW | 65 | GGATTAA(2-Deoxyuridine)AATGGAAGGTAATAGAGAAGATG | pYeDP60u-ura  pYeDP60u-leu |
| SmPIP11_Scu_RV | 65 | GGGTTAA(2-Deoxyuridine)TTAGTGAAATGGGATAGCTC |  |
| SmPIP21_Scu_FW | 64 | GGATTAA(2-Deoxyuridine)AATGTCAAAAGATTTGGAAAATGG |  |
| SmPIP21_Scu_RV | 65 | GGGTTAA(2-Deoxyuridine)TTAGTGCATAGAACTGGATC |  |
| SmPIP22_Scu_FW | 68 | GGATTAA(2-Deoxyuridine)AATGGCAAAAGATGCCTCTAAGG |  |
| SmPIP22_Scu_RV | 69 | GGGTTAA(2-Deoxyuridine)TTATGGGTGACCGTGATGTTG |  |
| SmPIP11_FW | 66 | GGCTTAA(2-Deoxyuridine)ATGGAAGGTAATAGAGAAGATG | pRS426-pTPI-N-ter-GFPu  pCAMBIA2300 35S N-ter mYFPu  pCAMBIA2300 35S N-ter mCFPu  pNB1u  pNB1YFPu |
| SmPIP11_RV | 64 | GGTTTAA(2-Deoxyuridine)TTAGTGAAATGGGATAGCTC |  |
| SmPIP21_FW | 65 | GGCTTAA(2-Deoxyuridine)ATGTCAAAAGATTTGGAAAATGG |  |
| SmPIP21_RV | 64 | GGTTTAA(2-Deoxyuridine)TTAGTGCATAGAACTGGATC |  |
| SmPIP22_FW | 69 | GGCTTAA(2-Deoxyuridine)ATGGCAAAAGATGCCTCTAAGG |  |
| SmPIP22_RV | 67 | GGTTTAA(2-Deoxyuridine)TTATGGGTGACCGTGATGTTG |  |
