# Supplementary material for: Heterotetramerization of Plant PIP1 and PIP2 Aquaporins Is an Evolutionary Ancient Feature to Guide PIP1 Plasma Membrane Localization and Function
Source: Front Plant Sci. 2018 Mar 26;9:382. doi: 10.3389/fpls.2018.00382 (PMC5879115; doi:10.3389/fpls.2018.00382)
Supplement: Supplementary file 2 [file Table_2.DOCX]

**Supplementary File 2:** Protein sequence alignment of SmPIPs together with ZmPIP1;2 and ZmPIP2;5, two typical representatives for PIP1 and PIP2-type isoforms. The four amino acid residues constituting the aromatic/arginine selectivity filters are highlighted in yellow. The ‘LxxxA’ motif residues are highlighted in magenta. Conserved amino acid residues which have been demonstrated to be of importance for post-translational regulatory processes and which are common to PIP1s and PIP2s are highlighted in green. NPA motifs are underlined. Diacidic motifs are displayed in bold blue letters.

SmPIP1_1 MEGNREDVHVGVAKYPERE-L---GTSAQAEKDYVEPPPTRLIEPSEFSS

SmPIP2_1 MSK**DLE**N--------------------GNAAKDFSEPPPTPLIDLAELKS

SmPIP2_2 MAKDASK----------------**ESE**AFVTAKDYEEPPPARLVDPKEFGS

ZmPIP1_2 MEGKEEDVRLGANKFSERQPIGTAAQGAADDKDYKEPPPAPLFEPGELKS

ZmPIP2_5 MAK**DIE**A------------------AAAHEGKDYSDPPPAPLVDAEELTK

* . . **: :***: *.: *: .

SmPIP1_1 WSFWRAGIAEFFATFLFLYITILTVVGNVDRT---------SCLGVGIQG

SmPIP2_1 WSLYRACIVELVATLLFLYIGVTALIGHARAQAA---AGGDSCGGIGLLG

SmPIP2_2 WSFYRAGIAEFVATLLFLYITVQTVIGHSRNA--------ANCGGVGLLG

ZmPIP1_2 WSFYRAGIAEFVATFLFLYITILTVMGVSKST--------SKCATVGIQG

ZmPIP2_5 WSLYRAVIAEFVATLLFLYITVATVIGYKHQTDAAASGPDAACGGVGVLG

**::** *.*:.**:***** : :::* * :*: *

SmPIP1_1 IAWAFGGMIFALVYCTAGISGGHINPAVTFGLFLARKVSLPRTLLYMVAQ

SmPIP2_1 VAWVFGGMIFVLVYCTAGVSGGHLNPAVTFGMFLARKVSIPRALLYVASQ

SmPIP2_2 IAWAFGGMIFVLVYCTAGISGGHINPAVTFGLFVARKVSLPRAIFYMIMQ

ZmPIP1_2 IAWSFGGMIFALVYCTAGISGGHINPAVTFGLFLARKLSLTRALFYIIMQ

ZmPIP2_5 IAWAFGGMIFILVYCTAGVSGGHINPAVTFGLFLARKVSLVRALLYIVAQ

:** ****** *******:****:*******:*:***:*: *:::*: *

SmPIP1_1 CLGAICGAGVVKGFQKAKFNAAGGGANYVH--HGYTIGDGLGAEIVGTFV

SmPIP2_1 VAGAIFGAGLAKGFQASFYNGNMGGATFIQ--NGYTKAEGLGAEIIGTFV

SmPIP2_2 CLGAIVGCGLAKGFQKSFYVQQGGGANSVARARGYSTGTGLGAEIIGTFV

ZmPIP1_2 CLGAVCGAGVVKGFQQGLYMGNGGGANVVA--PGYTKGDGLGAEIVGTFI

ZmPIP2_5 CLGAICGVGLVKGFQSAFYVRYGGGANELS--AGYSKGTGLAAEIIGTFV

**: * *:.**** . : ***. : **: . **.***:***:

SmPIP1_1 LVYTVFSATDAKRSARDSHVPLLAPLPIGFAVFLVHLATIPITGTGINPA

SmPIP2_1 LAYTVFSATDPKRVARDSHVPVLAPLPIGFAVFMVHLALLPVTGSSVNPA

SmPIP2_2 LVYTVFSATDPKRVARDSHVPVLAPLPIGFAVFMVHLATIPITGTGINPA

ZmPIP1_2 LVYTVFSATDAKRNARDSHVPILAPLPIGFAVFLVHLATIPITGTGINPA

ZmPIP2_5 LVYTVFSATDPKRNARDSHVPVLAPLPIGFAVFMVHLATIPITGTGINPA

*.********.** *******:***********:**** :*:**:.:***

SmPIP1_1 RSLGSAVIYNGDQAWDDHWIFWVGPLIGAALAAFYHQFVIRAIPF-----

SmPIP2_1 RSFATAVIYNNSRVWNDQWVYWVGPLLGAALAAMYHQYILRGGARAVRAL

SmPIP2_2 RSFGAAVIFNKSVSWDDQWIFWVGPFIGAAAAAIYHQYVLRAGSA-LKAL

ZmPIP1_2 RSLGAAIIYNRDHAWNDHWIFWVGPFIGAALAAIYHQVIIRAIPFKS---

ZmPIP2_5 RSLGAAVIYNNDKAWDDHWIFWVGPFIGAAIAAAYHQYVLRASAAKLGSS

**:.:*:*:* . *:*:*::****::*** ** *** ::*. .

SmPIP1_1 --------------H

SmPIP2_1 NSFRSSS------MH

SmPIP2_2 GSFRSNPPHQHHGHP

ZmPIP1_2 -------------RS

ZmPIP2_5 ASF----------SR
