# Supplementary material for: Heterotetramerization of Plant PIP1 and PIP2 Aquaporins Is an Evolutionary Ancient Feature to Guide PIP1 Plasma Membrane Localization and Function
Source: Front Plant Sci. 2018 Mar 26;9:382. doi: 10.3389/fpls.2018.00382 (PMC5879115; doi:10.3389/fpls.2018.00382)
Supplement: Supplementary file 3 [file Table_3.DOCX]

**Supplementary File S3:** Rate constants of SmPIP swelling kinetics. Rate constants were calculated based on the swelling kinetics detected by light scattering within the first second of swelling. To this aim traces were fitted to one- or two phase decay equations to obtain the best fit. Means of rate constant values for the fitted curves were determined by GraphPad Prism 6 and are displayed ± SD (n = 22-28).

| Constructs which are expressed in yeast spheroplasts | Rate constants (s^-1^) ± SD | Figure |
| --- | --- | --- |
| hAQP8 + ev | 111.0116 ± 46.41897813 | 3 B |
| ev + ev | 1.438104348 ± 0.325595532 | 3 B |
| SmPIP1;1 + SmPIP1,1 | 1.1071 ± 0.401472979 | 3 B |
| SmPIP1;1 + SmPIP2;1 | 42.56041667 ± 11.11029389 | 3 B |
| SmPIP1;1 + SmPIP2;2 | 1.213716 ± 0.213156567 | 3 B |
| SmPIP2;1 + SmPIP2;1 | 174.4392 ± 74.7651484 | 3 B |
| SmPIP2;1 + SmPIP2;2 | 42.23038462 ± 11.90015949 | 3 B |
| SmPIP2;2 + SmPIP2;2 | 1.129975 ± 0.442961026 | 3 B |
| SmPIP1;1 + ev | 1.765111111 ± 0.232601067 | 3 B |
| SmPIP2;2 + ev | 1.732333333 ± 0.309463431 | 3 B |
| SmPIP2;1 + ev | 53.13464286 ± 14.86439108 | 3 B |

| hAQP8 + ev | 99.95272727 ± 72.70390434 | 3 A |
| --- | --- | --- |
| ev + ev | 2.101678571 ± 0.42768568 | 3 A |
| GFP:SmPIP1;1 + ev | 1.599526923 ± 0.34802299 | 3 A |
| GFP:SmPIP1;1 + SmPIP2;1 | 63.8256 ± 23.52031021 | 3 A |
| GFP:SmPIP1;1 + SmPIP2;2 | 1.764423077 ± 0.394352753 | 3 A |
| GFP:SmPIP2;1 + ev | 33.5524 ± 10.9682315 | 3 A |
| GFP:SmPIP2;1 + SmPIP1;1 | 24.83217391 ± 9.174444227 | 3 A |
| GFP:SmPIP2;1 + SmPIP2;2 | 34.45384615 ± 7.365087414 | 3 A |
| GFP:SmPIP2;2 + ev | 1.683724 ± 0.572385196 | 3 A |
| GFP:SmPIP2;2 + PIP1;1 | 1.730512 ± 0.343761187 | 3 A |
| GFP:SmPIP2;2 + PIP;21 | 73.02826087 ± 32.83604251 | 3 A |
